# The CALLY Index as an integrated inflammation–nutrition biomarker for risk stratification in HFpEF

**DOI:** 10.1186/s12872-026-06300-y

**Published:** 2026-07-20

**Authors:** Jing Ge, Jinqi Liu, Yali Zhang, Dongdong Zhang, Shen Wu, Changjiang Xu, Dongying Zhang

**Affiliations:** 1 Department of Clinical Nutrition, The Second People’s Hospital of Huai’an /The Affiliated Huai’an Hospital of Xuzhou Medical University, Huai’an, Jiangsu Province China; 2 Department of Cardiology, The Second People’s Hospital of Huai’an /The Affiliated Huai’an Hospital of Xuzhou Medical University, Huai’an, Jiangsu Province China; 3 Department of Cardiology, Huai’an First People’s Hospital, Nanjing Medical University, No.1,West Huanghe Road, Huaiyin District, Huai’an, Jiangsu Province China

**Keywords:** Risk stratification, CRP–albumin–lymphocyte, Heart failure with preserved ejection fraction, Nutrition, Prognosis

## Abstract

**Background:**

Heart failure with preserved ejection fraction (HFpEF) is a complex systemic syndrome characterized by inflammation, metabolic dysregulation, and immune imbalance. However, simple and integrative biomarkers for risk stratification remain limited. The C-reactive protein–albumin–lymphocyte (CALLY) index, reflecting inflammation, nutritional status, and immune function, has shown prognostic value in various diseases, but its role in HFpEF remains unclear.

**Methods:**

In this single-center retrospective cohort study, 308 patients with HFpEF were enrolled. The CALLY index was calculated as albumin × lymphocyte count / CRP. The primary endpoint was major adverse cardiovascular events (MACE), defined as all-cause death or heart failure rehospitalization. Cox proportional hazards models, restricted cubic spline (RCS), receiver operating characteristic (ROC) analysis, and Kaplan–Meier curves were applied.

**Results:**

During follow-up, 87 patients (28.25%) experienced MACE. Multivariable Cox analysis identified the CALLY index (HR = 0.98, 95% CI: 0.96–0.99) and E/e′ (HR = 1.08, 95% CI: 1.01–1.17) as independent predictors of MACE. RCS analysis demonstrated a nonlinear inverse association between the CALLY index and MACE risk. ROC analysis showed good predictive performance (AUC = 0.82). Patients with lower CALLY index (< 11.8) had significantly higher event rates (log-rank *p* < 0.001).

**Conclusions:**

The CALLY index is an independent predictor of long-term outcomes in HFpEF, integrating inflammation, nutrition, and immune status. It may serve as a simple and practical tool for risk stratification.

## Introduction

Heart failure (HF) has become a major global health burden, with heart failure with preserved ejection fraction (HFpEF) accounting for more than half of all HF cases [1, 2]. Despite continuous advances in diagnostic criteria and management strategies, the long-term prognosis of HFpEF remains unsatisfactory [3]. Therefore, identifying simple and effective biomarkers that comprehensively reflect systemic inflammation, nutritional status, and immune function is of great clinical importance for early risk stratification and prognostic assessment.

With increasing recognition of its complex nature, HFpEF is no longer regarded as a purely “diastolic” disorder but rather as a systemic syndrome characterized by multisystem abnormalities [4, 5]. Accumulating evidence indicates that chronic low-grade inflammation plays a central role in the pathogenesis and progression of HFpEF. Inflammatory mediators such as C-reactive protein (CRP), interleukin-6 (IL-6), and tumor necrosis factor-α (TNF-α) are significantly elevated in HFpEF patients and contribute to endothelial dysfunction, myocardial fibrosis, and impaired calcium handling, ultimately leading to diastolic dysfunction and increased myocardial stiffness [6]. In addition, malnutrition and immune dysregulation are important determinants of adverse outcomes in HFpEF [7].

Traditional inflammatory markers, including CRP, neutrophil-to-lymphocyte ratio (NLR), and platelet-to-lymphocyte ratio (PLR), can partially reflect inflammatory activity but are susceptible to confounding factors such as infection, medications, and acute stress, thereby limiting their predictive value. Similarly, nutritional indicators such as albumin, prealbumin, and body mass index mainly reflect long-term nutritional reserves but fail to capture the interplay between inflammation and immune status [8]. Therefore, single biomarkers are insufficient to characterize the complex pathophysiological profile of HFpEF.

The C-reactive protein–albumin–lymphocyte (CALLY) index is a novel composite biomarker that integrates inflammation, nutritional status, and immune function. Previous studies have demonstrated its prognostic value in various conditions, including cancer and sepsis [9, 10]. Moreover, emerging evidence suggests that the CALLY index is significantly associated with cardiovascular outcomes, highlighting its potential utility in cardiovascular risk assessment [11]. However, its role in HFpEF remains largely unexplored.

Given the pivotal roles of inflammation and nutritional imbalance in HFpEF, investigating integrative biomarkers that capture multiple physiological dimensions is of considerable clinical relevance. Therefore, this study aimed to systematically evaluate the prognostic value of the CALLY index in patients with HFpEF, with a particular focus on its ability to predict long-term major adverse cardiovascular events (MACE), in order to provide a simple and practical tool for risk stratification.

## Materials and methods

### Study population

This was a single-center, retrospective observational study. Consecutive patients hospitalized in the Department of Cardiology at Huai’an Second People’s Hospital between June 2022 and December 2025, who were diagnosed with heart failure with preserved ejection fraction (HFpEF), were enrolled. The study protocol was reviewed and approved by the Ethics Committee of Huai’an Second People’s Hospital (approval No.HEYLL202644). Given the retrospective and observational nature of the study, which involved only the collection of existing clinical data without any additional intervention, informed consent was waived in accordance with institutional regulations. The study was conducted in strict compliance with the principles of the Declaration of Helsinki and relevant ethical guidelines. The diagnosis of HFpEF was based on the criteria recommended by the European Society of Cardiology (ESC) guidelines [12]. Specifically, HFpEF was defined by the presence of clinical manifestations of acute heart failure, along with the following criteria: (1) a left ventricular ejection fraction (LVEF) ≥ 50%; and (2) at least one objective indicator of heart failure, such as elevated N-terminal pro–B-type natriuretic peptide (NT-proBNP) levels, or evidence of cardiac structural or functional abnormalities on echocardiography. The inclusion criteria were as follows: (1) age ≥ 18 years; (2) a confirmed diagnosis of HFpEF; and (3) complete clinical data. The exclusion criteria were: (1) acute myocardial infarction during hospitalization, acute decompensated pulmonary edema requiring mechanical ventilation, or severe valvular heart disease with significant hemodynamic impact (including severe aortic stenosis, severe aortic regurgitation, severe mitral stenosis, severe mitral regurgitation, or severe tricuspid regurgitation); (2) active infection or autoimmune disease (including pneumonia, acute exacerbation of chronic obstructive pulmonary disease, sepsis, systemic inflammatory disease, or current immunosuppressive therapy); (3) malignancy or severe hepatic or renal dysfunction (estimated glomerular filtration rate [eGFR] < 30 mL/min/1.73 m² or Child–Pugh class C cirrhosis); and (4) loss to follow-up.

**Fig. 1:**
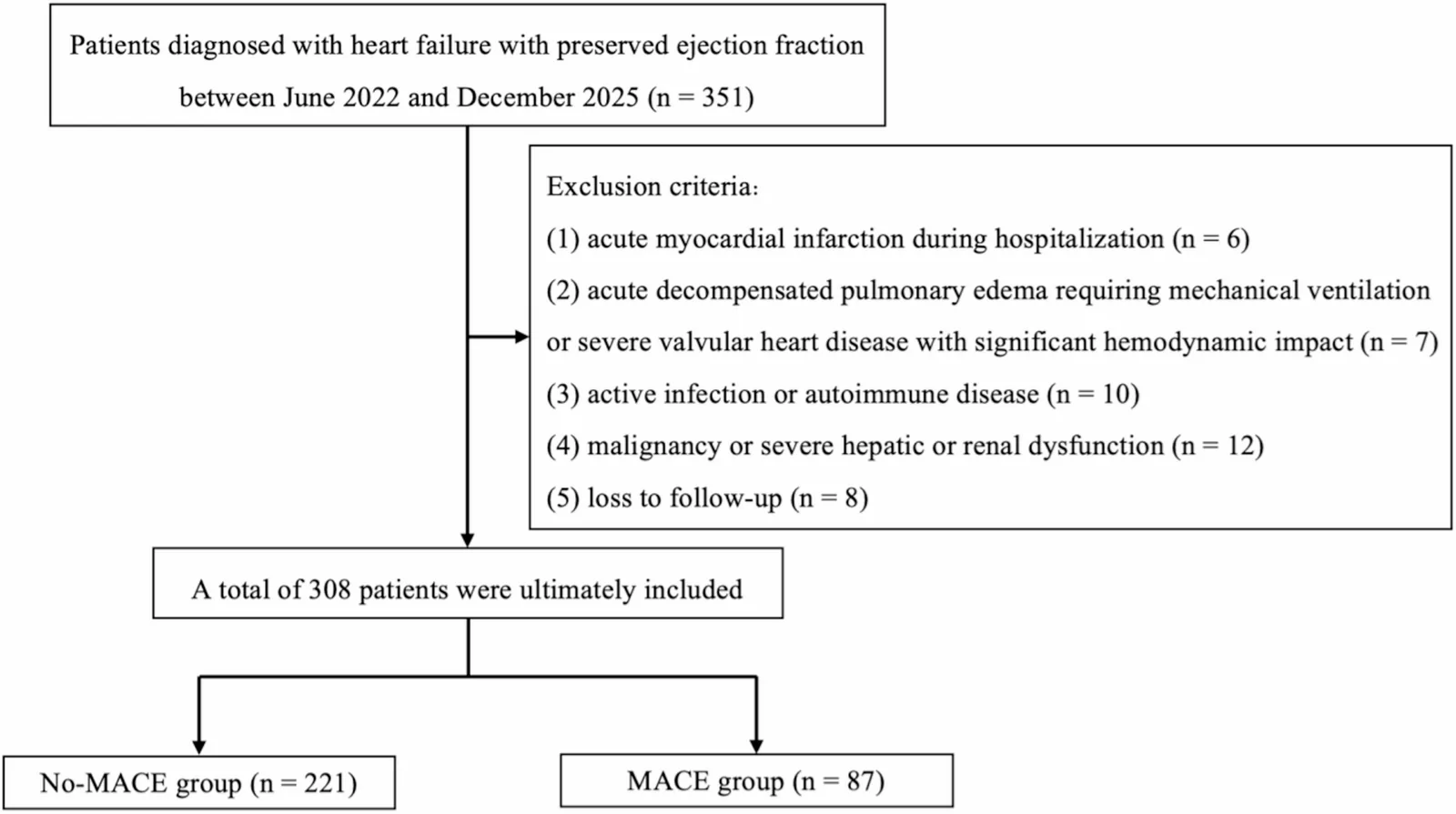
Study flowchart. MACE, major adverse cardiovascular events

After applying the predefined inclusion and exclusion criteria, a total of 308 patients with HFpEF were ultimately included in the final analysis. The patient selection process is illustrated in Fig. 1.

### Data collection and variable definitions

Clinical data of all enrolled patients were retrospectively obtained from the hospital electronic medical record (EMR) system. The collected variables included the following categories: Age, sex, body mass index (BMI), smoking status, and medical history including hypertension and diabetes mellitus. All laboratory parameters were obtained from the first venous blood samples collected within 24 h of admission. These included complete blood count (white blood cell count, neutrophil count, lymphocyte count, hemoglobin, and platelet count), liver and renal function, estimated glomerular filtration rate (eGFR), fasting plasma glucose, and lipid profile (total cholesterol [TC], triglycerides [TG], low-density lipoprotein cholesterol [LDL-C], high-density lipoprotein cholesterol [HDL-C], and lipoprotein(a)). In addition, high-sensitivity C-reactive protein (hs-CRP), uric acid (UA), and N-terminal pro–B-type natriuretic peptide (NT-proBNP) were measured. CALLY index, a composite biomarker integrating nutritional status, immune function, and inflammatory response, was calculated as follows [9–11, 13]: CALLY index = (albumin [g/L] × lymphocyte count [×10⁹/L]) / CRP (mg/L). All patients underwent transthoracic echocardiography within 48 h of admission. The following parameters were obtained: left ventricular ejection fraction (LVEF), measured using the modified biplane Simpson method; left atrial volume index (LAVI), calculated using the area–length method at end-systole and indexed to body surface area; left ventricular mass index (LVMI), calculated using the Devereux formula and indexed to body surface area; relative wall thickness (RWT), defined as 2 × posterior wall thickness / left ventricular end-diastolic diameter; peak early (E) and late (A) diastolic mitral inflow velocities. Information on medications prescribed during hospitalization and at discharge was recorded, including diuretics, β-blockers, angiotensin-converting enzyme inhibitors (ACEIs), angiotensin II receptor blockers (ARBs), and statins.

### Follow-up and outcome definitions

All enrolled patients were systematically followed from the date of hospital discharge. Follow-up was conducted through outpatient visits, structured telephone interviews, and review of the electronic medical record system. Information collected during follow-up included survival status, rehospitalization, and the occurrence of cardiovascular events. The primary endpoint was major adverse cardiovascular events (MACE), defined as the first occurrence of either all-cause mortality or heart failure rehospitalization during the follow-up period. All-cause mortality was defined as death due to any cause, including both cardiovascular and non-cardiovascular causes. The diagnosis of heart failure was based on the latest ESC guidelines [12]. For patients experiencing multiple events during follow-up, only the first event was considered for analysis, and subsequent events were not included.

### Statistical analysis

All statistical analyses were performed using SPSS version 27.0 (IBM Corporation, Armonk, NY, USA) and R version 4.3.2 (R Foundation for Statistical Computing, Vienna, Austria). All tests were two-sided, and a P value < 0.05 was considered statistically significant. The normality of continuous variables was assessed using the Shapiro–Wilk test. Continuous variables with a normal distribution were expressed as mean ± standard deviation (SD) and compared using the independent samples t-test. Non-normally distributed variables were presented as median (interquartile range, IQR) and compared using the Mann–Whitney U test. Categorical variables were expressed as counts (percentages) and compared using the chi-square test. The association between the CALLY index and long-term risk of major adverse cardiovascular events (MACE) in patients with HFpEF was evaluated using Cox proportional hazards regression models. Initially, univariable Cox regression analyses were performed to identify potential predictors of MACE. BMI and variables with *P* < 0.10 in univariable analyses were considered candidates for inclusion in the multivariable model. Subsequently, multivariable Cox regression models were constructed using a stepwise adjustment strategy to assess the independent prognostic value of the CALLY index. The proportional hazards assumption was tested using Schoenfeld residuals. To explore potential nonlinear dose–response relationships between the CALLY index and MACE risk, restricted cubic spline (RCS) regression analysis was performed within the multivariable Cox model framework. Receiver operating characteristic (ROC) curve analysis was used to evaluate the discriminative ability of the CALLY index for predicting MACE. The area under the curve (AUC) and corresponding 95% confidence interval (CI) were calculated. The optimal cut-off value of the CALLY index was determined using the Youden index. Based on the optimal cut-off value derived from ROC analysis, patients were categorized into high and low CALLY groups. To assess the incremental predictive value of the CALLY index, a baseline clinical model was constructed using age, NT-proBNP, E/e′, LVMI, and neutrophil count. The performance of the baseline model was compared with that of a model additionally incorporating the CALLY index. Reclassification and discrimination improvement were further assessed using continuous net reclassification improvement (NRI) and integrated discrimination improvement (IDI). Kaplan–Meier survival curves were constructed to estimate event-free survival, and differences between groups were compared using the log-rank test.

## Results

### Baseline characteristics

A total of 308 patients with HFpEF were included in the final analysis. During a median follow-up of 17 (9, 29) months, 87 patients (28.25%) experienced MACE, including 32 cases of all-cause mortality and 55 cases of heart failure rehospitalization, while 221 patients (71.75%) remained event-free. The mean age of the overall cohort was 69.28 ± 13.94 years, and 189 patients (61.36%) were male.

Detailed baseline characteristics of the study population are presented in Table 1. In terms of demographic and clinical characteristics, patients in the MACE group were significantly older than those in the non-MACE group (71.93 ± 12.51 vs. 68.23 ± 14.35 years, *P* = 0.036). Regarding laboratory parameters, compared with the non-MACE group, patients in the MACE group had significantly lower estimated glomerular filtration rate (eGFR) (99.48 ± 25.25 vs. 106.56 ± 18.41 mL/min/1.73 m², *P* = 0.019), lower serum albumin levels (40.64 ± 4.21 vs. 42.09 ± 4.95 g/L, *P* = 0.016), and lower lymphocyte counts (1.28 ± 0.57 vs. 1.86 ± 1.24 × 10⁹/L, *P* < 0.001). In contrast, neutrophil counts (8.66 ± 2.53 vs. 7.43 ± 2.79 × 10⁹/L, *P* < 0.001), C-reactive protein (CRP) levels (7.15 ± 3.81 vs. 3.32 ± 3.22 mg/L, *P* < 0.001), and NT-proBNP levels [1354.52 (240.28, 3390.21) vs. 470.50 (185.36, 1510.21) pg/mL, *P* < 0.001] were significantly higher in the MACE group. Notably, the CALLY index was significantly lower in patients who developed MACE compared with those who did not [7.29 (4.60, 11.30) vs. 30.49 (11.99, 83.82), *P* < 0.001]. With respect to echocardiographic parameters, the E/e′ ratio was significantly higher in the MACE group than in the non-MACE group (15.34 ± 3.14 vs. 14.46 ± 3.13, *P* = 0.027), indicating more severe left ventricular diastolic dysfunction in patients who experienced adverse events.

**Table 1:** Baseline characteristics of all patients

Variables	Total (*n* = 308)	No MACE (*n* = 221)	MACE (*n* = 87)	*P* value
Age (years)	69.28 ± 13.94	68.23 ± 14.35	71.93 ± 12.51	0.036
BMI (kg/m²)	25.23 ± 3.49	25.21 ± 3.63	25.27 ± 3.13	0.902
SBP (mmHg)	132.12 ± 19.46	131.50 ± 19.11	133.70 ± 20.35	0.372
DBP (mmHg)	73.80 ± 13.24	74.17 ± 13.35	72.84 ± 12.99	0.427
Heart rate (bpm)	75.92 ± 14.74	76.36 ± 15.42	74.79 ± 12.88	0.401
Male, n (%)	189 (61.36)	142 (64.25)	47 (54.02)	0.097
Smoking, n (%)	40 (12.99)	31 (14.03)	9 (10.34)	0.387
Diabetes, n (%)	96 (31.17)	63 (28.51)	33 (37.93)	0.108
Hypertension, n (%)	219 (71.10)	151 (68.33)	68 (78.16)	0.086
CALLY index	18.08 (7.90, 65.09)	30.49 (11.99, 83.82)	7.29 (4.60, 11.30)	< 0.001
Albumin (g/L)	41.68 ± 4.79	42.09 ± 4.95	40.64 ± 4.21	0.016
White blood cell count (×10⁹/L)	10.02 ± 2.91	9.86 ± 3.02	10.42 ± 2.59	0.129
Neutrophil count (×10⁹/L)	7.78 ± 2.77	7.43 ± 2.79	8.66 ± 2.53	< 0.001
Lymphocyte count (×10⁹/L)	1.70 ± 1.12	1.86 ± 1.24	1.28 ± 0.57	< 0.001
Monocyte count (×10⁹/L)	0.54 ± 0.26	0.54 ± 0.25	0.53 ± 0.29	0.721
NT-proBNP (pg/mL)	604.2 (196.8, 2112.8)	470.5 (185.4, 1510.2)	1354.5 (240.3, 3390.2)	< 0.001
Lipoprotein(a) (mg/L)	111.6 (67.2, 219.2)	111.2 (65.7, 213.8)	122.3 (77.3, 248.5)	0.150
CRP (mg/L)	4.40 ± 3.81	3.32 ± 3.22	7.15 ± 3.81	< 0.001
Hemoglobin (g/L)	129.45 ± 22.47	130.72 ± 22.96	126.22 ± 20.94	0.113
Fasting glucose (mmol/L)	6.70 ± 2.98	6.85 ± 3.33	6.32 ± 1.79	0.158
Triglycerides (mmol/L)	1.64 ± 1.42	1.71 ± 1.57	1.44 ± 0.90	0.130
Total cholesterol (mmol/L)	4.01 ± 1.25	4.03 ± 1.32	3.96 ± 1.05	0.660
LDL-C (mmol/L)	2.18 ± 1.06	2.19 ± 1.09	2.16 ± 0.96	0.814
HDL-C (mmol/L)	1.14 ± 0.30	1.13 ± 0.30	1.19 ± 0.29	0.095
Uric acid (µmol/L)	321.43 ± 103.83	322.09 ± 109.80	319.75 ± 87.37	0.859
HbA1c (%)	6.56 ± 1.22	6.58 ± 1.27	6.50 ± 1.08	0.615
eGFR (mL/min/1.73 m²)	104.56 ± 20.78	106.56 ± 18.41	99.48 ± 25.25	0.019
RWT	0.42 ± 0.11	0.42 ± 0.13	0.42 ± 0.08	0.731
LVMI (g/m²)	99.74 ± 37.85	97.26 ± 34.50	106.04 ± 44.88	0.067
E/e′	14.71 ± 3.15	14.46 ± 3.13	15.34 ± 3.14	0.027
LAVI (mL/m²)	42.55 ± 6.22	42.71 ± 6.42	42.13 ± 5.67	0.458
LVEF (%)	64.72 ± 7.05	64.81 ± 6.82	64.52 ± 7.66	0.747
LA diameter (mm)	39.20 ± 6.54	39.42 ± 6.82	38.64 ± 5.77	0.351
LVD (mm)	46.93 ± 7.01	46.71 ± 6.14	47.49 ± 8.86	0.378
LVS (mm)	31.03 ± 7.99	30.75 ± 6.92	31.74 ± 10.24	0.331
IVS (mm)	11.03 ± 2.22	10.94 ± 2.29	11.28 ± 2.01	0.228
LVPW (mm)	9.59 ± 1.55	9.52 ± 1.62	9.79 ± 1.35	0.158
PA (mm)	21.18 ± 3.31	21.37 ± 3.48	20.71 ± 2.81	0.119
Statins, n (%)	185 (60.06)	135 (61.09)	50 (57.47)	0.560
Diuretics, n (%)	137 (44.48)	100 (45.25)	37 (42.53)	0.665
ACEI/ARB, n (%)	157 (50.97)	116 (52.49)	41 (47.13)	0.397
β-blockers, n (%)	203 (65.91)	140 (63.35)	63 (72.41)	0.131

*CALLY* Creactive protein–albumin–lymphocyte. *MACE *major adverse cardiovascular events, *BMI* body mass index, *SBP *systolic blood pressure, *DBP *diastolic blood pressure, *CRP* C-reactive protein, *NT-proBNP *N-terminal pro–B-type natriuretic peptide, *LDL-C* low-density lipoprotein cholesterol, *HDL-C* high-density lipoprotein cholesterol, *eGFR* estimated glomerular filtration rate, *LVMI *left ventricular mass index, *LAVI *eft atrial volume index, *LVEF* left ventricular ejection fraction, *RWT* relative wall thickness, *LA* left atrium, *LVD* left ventricular diameter, *LVS* left ventricular systolic diameter, *IVS* interventricular septal thickness, *LVPW* left ventricular posterior wall thickness, *PA* pulmonary artery diameter, *ACEI* angiotensin-converting enzyme inhibitor, *ARB* angiotensin II receptor blocker

### Cox proportional hazards regression analysis of the association between the CALLY index and MACE risk

The results of the univariable and multivariable Cox proportional hazards regression analyses are presented in Table 2. In the univariable Cox analysis, NT-proBNP (HR = 1.48, 95% CI: 1.05–2.09, *P* = 0.025), left ventricular mass index (LVMI) (HR = 1.01, 95% CI: 1.01–1.01, *P* = 0.017), E/e′ ratio (HR = 1.09, 95% CI: 1.02–1.17, *P* = 0.016), CALLY index (HR = 0.97, 95% CI: 0.96–0.99, *P* < 0.001), albumin (HR = 0.93, 95% CI: 0.89–0.98, *P* = 0.004), C-reactive protein (CRP) (HR = 1.18, 95% CI: 1.12–1.24, *P* < 0.001), neutrophil count (HR = 1.12, 95% CI: 1.04–1.21, *P* = 0.002), and lymphocyte count (HR = 0.57, 95% CI: 0.41–0.78, *P* < 0.001) were significantly associated with the risk of MACE.

BMI and variables with *P* < 0.10 in the univariable analysis were subsequently entered into the multivariable Cox regression model. After adjustment for these covariates, the CALLY index remained an independent protective predictor of MACE (adjusted HR = 0.98, 95% CI: 0.96–0.99, *P* < 0.001). In addition, the E/e′ ratio was identified as an independent risk factor for MACE (adjusted HR = 1.08, 95% CI: 1.01–1.17, *P* = 0.029).

**Table 2:** Univariable and multivariable cox proportional hazards regression analysis for MACE

Variables	Univariate		Multivariate	
	*P*	HR (95%CI)	*P*	HR (95%CI)
Male	0.893	1.03 (0.67 ~ 1.58)		
Smoking	0.438	1.32 (0.66 ~ 2.64)		
Diabetes	0.091	1.46 (0.94 ~ 2.25)		
Hypertension	0.074	1.59 (0.96 ~ 2.65)		
Statins	0.376	0.83 (0.54 ~ 1.26)		
Diuretics	0.651	0.91 (0.59 ~ 1.39)		
ACEI/ARB	0.480	0.86 (0.56 ~ 1.31)		
β-blockers	0.414	1.22 (0.76 ~ 1.95)		
Age	0.064	1.02 (1.00 ~ 1.03)		
BMI	0.985	1.00 (0.94 ~ 1.06)		
SBP	0.882	1.00 (0.99 ~ 1.01)		
DBP	0.976	1.00 (0.98 ~ 1.02)		
Heart rate	0.412	0.99 (0.98 ~ 1.01)		
NT-proBNP	0.025	1.48 (1.05 ~ 2.09)		
Hemoglobin	0.696	1.00 (0.99 ~ 1.01)		
Fasting glucose	0.446	0.96 (0.88 ~ 1.06)		
Triglycerides	0.174	0.85 (0.67 ~ 1.08)		
Total cholesterol	0.404	0.93 (0.79 ~ 1.10)		
LDL-C	0.578	0.94 (0.77 ~ 1.16)		
HDL-C	0.339	1.35 (0.73 ~ 2.52)		
Uric acid	0.299	1.00 (1.00 ~ 1.00)		
Lipoprotein(a)	0.243	1.41 (0.79 ~ 2.52)		
HbA1c	0.296	0.91 (0.77 ~ 1.08)		
eGFR	0.074	0.99 (0.98 ~ 1.00)		
RWT	0.627	1.53 (0.28 ~ 8.42)		
LVMI	0.017	1.01 (1.01 ~ 1.01)		
E/e′	0.016	1.09 (1.02 ~ 1.17)	0.029	1.08 (1.01 ~ 1.17)
LAVI	0.653	0.99 (0.96 ~ 1.03)		
LVEF	0.530	0.99 (0.96 ~ 1.02)		
LA	0.508	0.99 (0.96 ~ 1.02)		
LVD	0.254	1.02 (0.99 ~ 1.05)		
LVS	0.116	1.02 (0.99 ~ 1.05)		
IVS	0.149	1.06 (0.98 ~ 1.16)		
LVPW	0.111	1.10 (0.98 ~ 1.24)		
PA	0.253	0.96 (0.89 ~ 1.03)		
CALLY index	< 0.001	0.97 (0.96 ~ 0.99)	< 0.001	0.98 (0.96 ~ 0.99)
Albumin	0.004	0.93 (0.89 ~ 0.98)		
CRP	< 0.001	1.18 (1.12 ~ 1.24)		
White blood cell count	0.152	1.05 (0.98 ~ 1.14)		
Neutrophil count	0.002	1.12 (1.04 ~ 1.21)		
Lymphocyte count	< 0.001	0.57 (0.41 ~ 0.78)		
Monocyte count	0.696	0.85 (0.38 ~ 1.91)		

*CALLY *Creactive protein–albumin–lymphocyte, *MACE *major adverse cardiovascular events, *BMI *body mass index, *SBP *systolic blood pressure, *DBP *diastolic blood pressure, *CRP *C-reactive protein, *NT-proBNP* N-terminal pro–B-type natriuretic peptide, *LDL-C* low-density lipoprotein cholesterol, *HDL-C* high-density lipoprotein cholesterol, *eGFR* estimated glomerular filtration rate, *LVMI* left ventricular mass index, *LAVI* left atrial volume index, *LVEF* left ventricular ejection fraction, *RWT* relative wall thickness, *LA *left atrium, *LVD *left ventricular diameter, *LVS *left ventricular systolic diameter, *IVS *interventricular septal thickness, *LVPW *left ventricular posterior wall thickness, *PA *pulmonary artery diameter, *ACEI *angiotensin-converting enzyme inhibitor, *ARB *angiotensin II receptor blocker

### Restricted cubic spline analysis of the association between the CALLY index and MACE risk

**Fig. 2:**
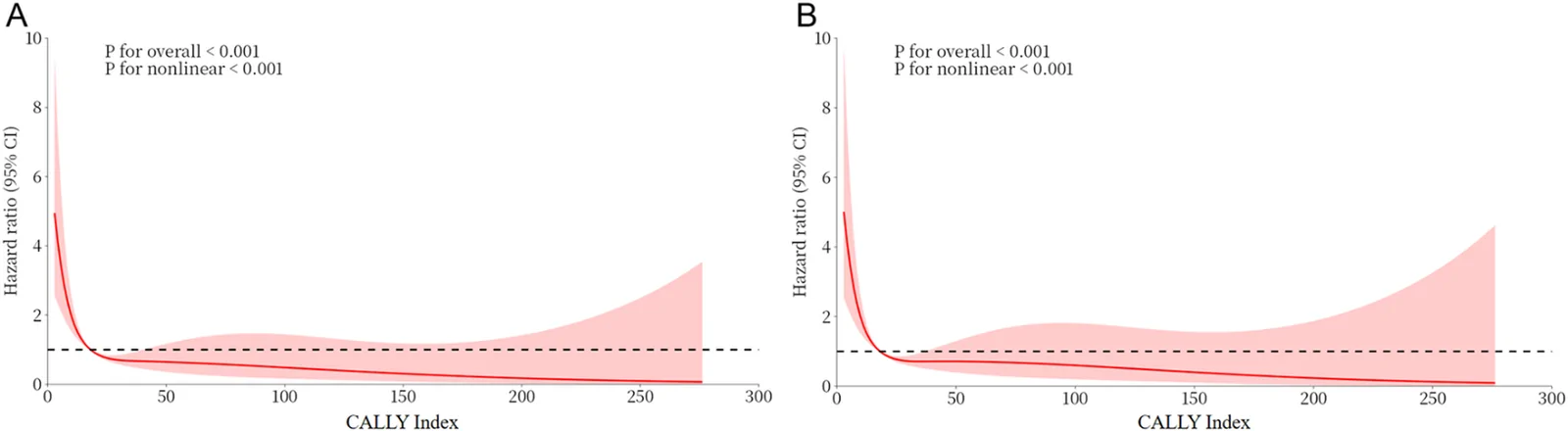
Restricted cubic spline analysis of the association between the CALLY index and risk of MACE. **A** Unadjusted model. **B** Multivariable-adjusted model. CALLY, Creactive protein–albumin–lymphocyte; MACE, major adverse cardiovascular events

To further investigate the dose–response relationship between the CALLY index and the risk of MACE, restricted cubic spline (RCS) analyses were performed within the framework of Cox regression models (Fig. 2A and B). The results demonstrated a significant overall association between the CALLY index and MACE risk (Poverall < 0.001), as well as a significant nonlinear relationship (Pnon-linearity < 0.001). Consistent nonlinear trends were observed in both the unadjusted model (Fig. 2A) and the multivariable-adjusted model (Fig. 2B). Specifically, the hazard ratio (HR) for MACE decreased progressively with increasing CALLY index. This decline was steeper at lower levels of the CALLY index and became more gradual at higher levels, suggesting a diminishing marginal effect.

### Predictive performance of the CALLY index for MACE: ROC curve analysis

**Fig. 3:**
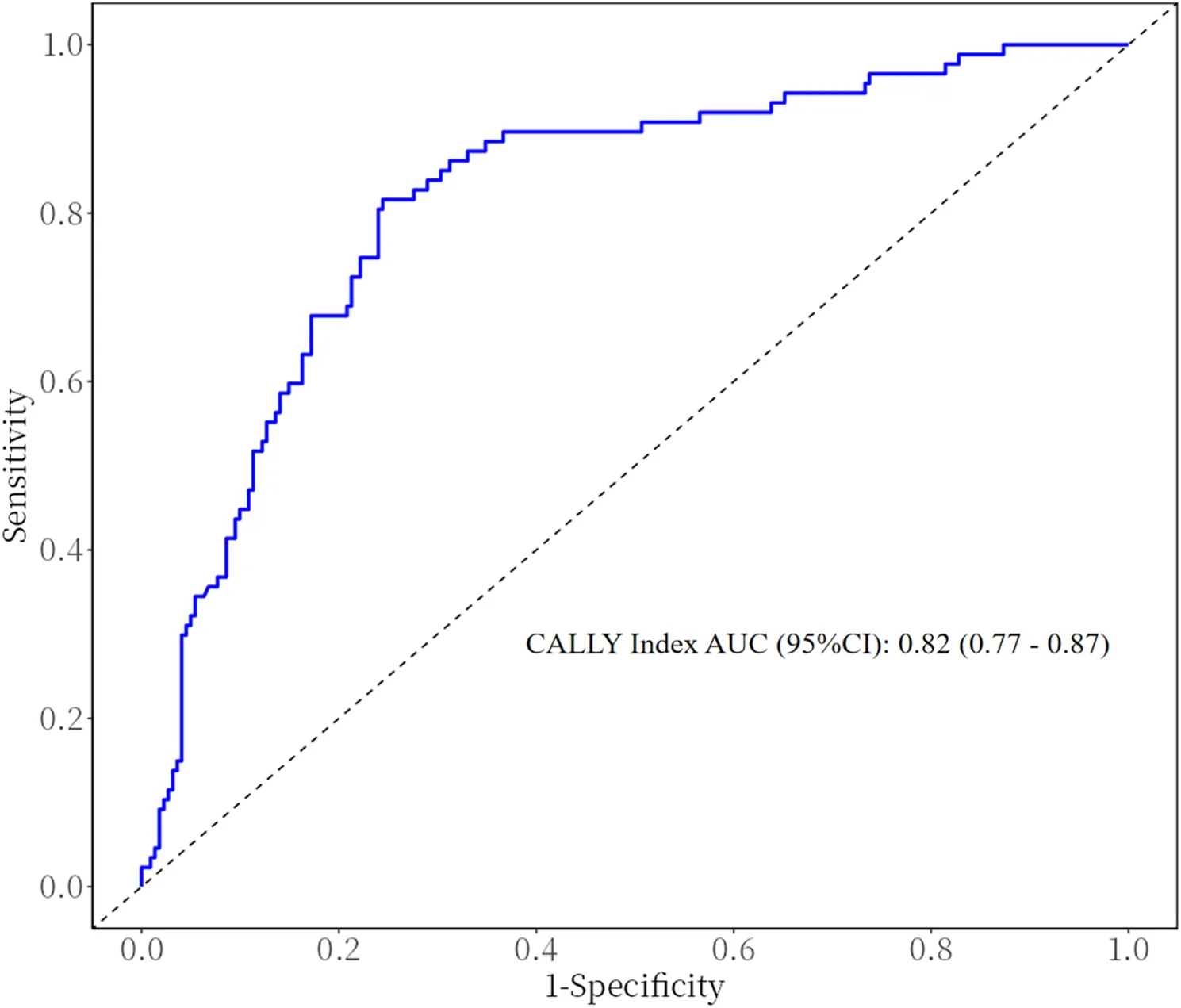
Receiver operating characteristic (ROC) curve of the CALLY index for predicting MACE

The results of the receiver operating characteristic (ROC) curve analysis are shown in Fig. 3. The CALLY index demonstrated good discriminative ability for predicting MACE, with an area under the curve (AUC) of 0.82 (95% CI: 0.77–0.87), indicating a strong capacity to identify adverse outcomes in patients with HFpEF. According to the Youden index, the optimal cut-off value of the CALLY index for predicting MACE was 11.8. At this threshold, the sensitivity and specificity were 0.76 and 0.82, respectively, suggesting potentially useful discriminative performance in this cohort.

### Incremental predictive value of the CALLY index beyond the clinical model

To further evaluate the incremental predictive value of the CALLY index, NRI and IDI analyses were performed by comparing the clinical model with and without the CALLY index. The clinical model included age, NT-proBNP, E/e′, LVMI, and neutrophil count. The addition of the CALLY index significantly improved risk reclassification and discrimination, with an NRI of 0.906 (95% CI: 0.555–1.210, *P* < 0.001) and an IDI of 0.148 (95% CI: 0.077–0.262, *P* < 0.001) (Table 3).

**Table 3:** Incremental predictive value of the CALLY index

Models	NRI	95% CI	*P*	IDI	95% CI	*P*
Clinical model	Reference	-	-	Reference	-	-
Clinical model + CALLY index	0.906	0.555 ~ 1.210	< 0.001	0.148	0.077 ~ 0.262	< 0.001

*CALLY* C-reactive protein–albumin–lymphocyte, *NRI *net reclassification improvement, *IDI *integrated discrimination improvement, *NT-proBNP* N-terminal pro–B-type natriuretic peptide, *LVMI* left ventricular mass index

### Kaplan–Meier survival analysis

**Fig. 4:**
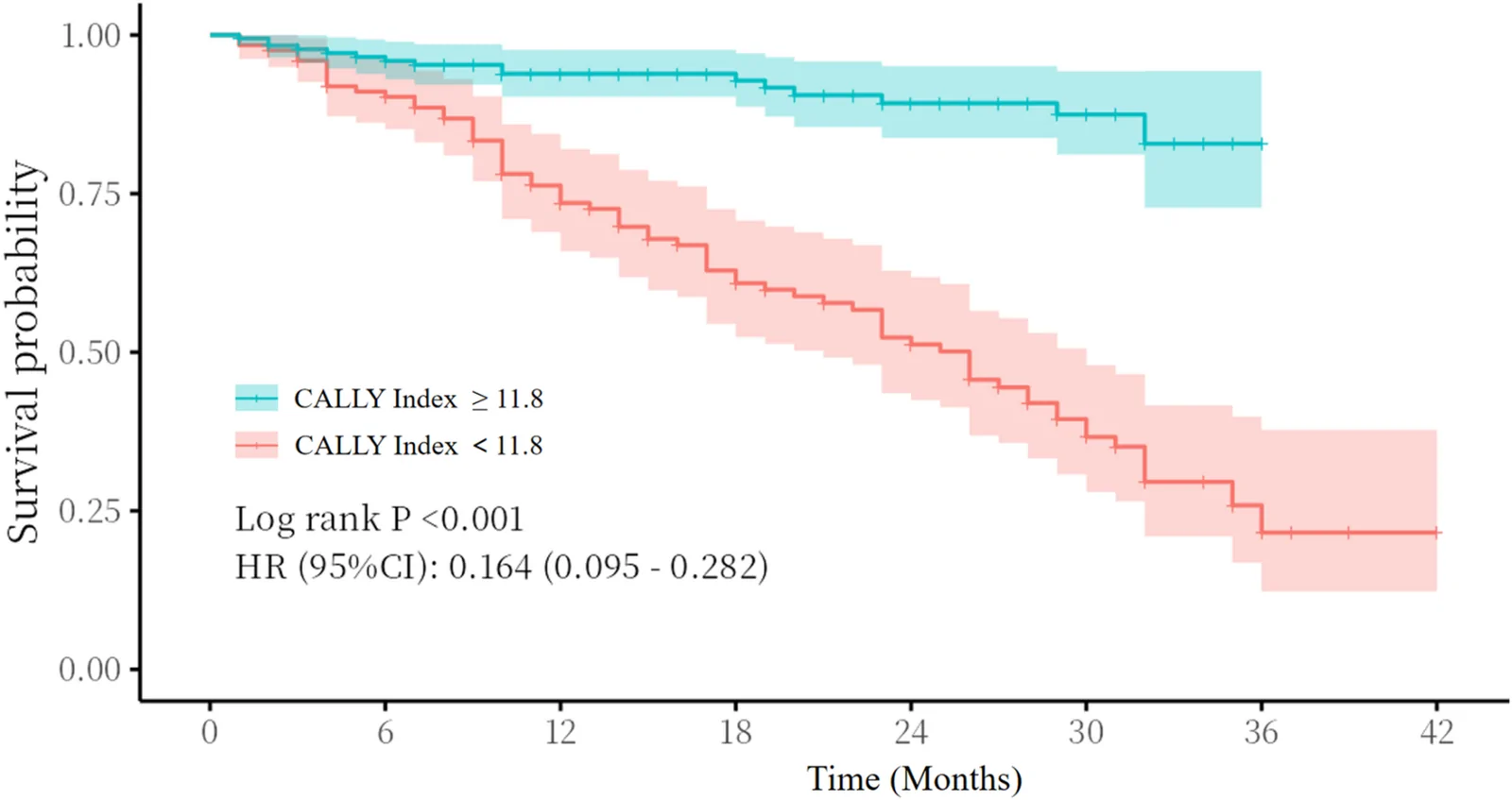
Kaplan–Meier curves for MACE-free survival according to CALLY index

Based on the optimal cut-off value of 11.8 derived from ROC analysis, the 308 patients were categorized into a high CALLY group (CALLY index ≥ 11.8) and a low CALLY group (CALLY index < 11.8). The Kaplan–Meier survival curves (Fig. 4) demonstrated a significant difference in MACE-free survival between the two groups during follow-up (log-rank *P* < 0.001). Specifically, the cumulative incidence of MACE was significantly higher in the low CALLY group compared with the high CALLY group. Patients in the low CALLY group had a markedly increased risk of MACE compared with those in the high CALLY group (HR = 0.164, 95% CI: 0.095–0.282). For clarity, a higher CALLY index was associated with a substantially lower risk of adverse cardiovascular events.

## Discussion

The present study systematically evaluated the prognostic value of the CALLY index in patients with HFpEF. The main findings can be summarized as follows. First, the CALLY index was significantly associated with the risk of long-term MACE in patients with HFpEF and remained an independent protective factor after multivariable adjustment. Second, RCS analysis revealed a significant nonlinear association, demonstrating a clear dose–response relationship between the CALLY index and MACE risk. Third, the CALLY index showed good discriminative ability in ROC analysis (AUC = 0.82), with high specificity. Finally, Kaplan–Meier analysis further confirmed that patients with lower CALLY index values had a significantly higher risk of adverse events. Taken together, these findings consistently support the potential clinical utility of the CALLY index for risk stratification in HFpEF.

HFpEF is a highly heterogeneous syndrome characterized by the interplay of multiple pathophysiological mechanisms, including chronic low-grade inflammation, endothelial dysfunction, myocardial remodeling, and metabolic disturbances [14–17]. Unlike heart failure with reduced ejection fraction (HFrEF), HFpEF is closely associated with metabolic abnormalities, systemic inflammation, and microvascular dysfunction [18]. In this context, inflammation and nutritional status have emerged as critical determinants of prognosis in HFpEF. Previous studies have shown that elevated CRP levels are associated with increased risk of heart failure hospitalization and mortality [19], while hypoalbuminemia reflects both inflammatory consumption and malnutrition and is an independent predictor of adverse outcomes [20]. In addition, lymphopenia has been linked to immune suppression and neurohormonal activation, and is associated with disease severity and mortality risk [21]. However, these single biomarkers capture only isolated aspects of the disease process and are insufficient to fully characterize the complex inflammation–nutrition–immune network in HFpEF.

The CALLY index integrates CRP, albumin, and lymphocyte count, thereby providing a comprehensive assessment of inflammatory burden, nutritional reserve, and immune status. Previous studies have demonstrated its prognostic value in patients with cancer, sepsis, and acute coronary syndromes [22, 23]. However, its role in HFpEF, a heterogeneous syndrome frequently accompanied by inflammation, metabolic abnormalities, and multimorbidity, has not been systematically explored. The present study fills this gap and further demonstrates that the CALLY index is not only significantly associated with adverse outcomes but also exhibits good discriminative performance (AUC = 0.82), highlighting its potential clinical applicability in risk assessment. Notably, this study is the first to demonstrate a nonlinear association between the CALLY index and MACE risk using RCS analysis. Specifically, at lower levels of the CALLY index, the risk of MACE decreased sharply with increasing values, whereas at higher levels, the decline in risk became more gradual. This pattern suggests a diminishing marginal effect and indicates the presence of a potential threshold range. In other words, patients with low CALLY index values may represent a clinically vulnerable subgroup characterized by systemic inflammation, impaired nutritional status, immune dysregulation, frailty, and multimorbidity. These patients may be more sensitive to improvements in the CALLY index and could potentially derive greater benefit from targeted interventions. This finding provides a rationale for future studies exploring CALLY-guided precision management strategies.

From a mechanistic perspective, the association between the CALLY index and adverse outcomes may be explained by the synergistic effects of inflammation, malnutrition, and immune dysfunction. Elevated CRP reflects persistent inflammatory activation, which can promote myocardial hypertrophy, interstitial fibrosis, and microvascular dysfunction through pathways involving NF-κB, IL-6, and TNF-α, ultimately leading to impaired diastolic function [6, 24]. Hypoalbuminemia not only indicates poor nutritional status but also contributes to reduced oncotic pressure, impaired microcirculatory perfusion, and diminished antioxidant capacity, thereby exacerbating myocardial metabolic dysfunction [25, 26]. Meanwhile, lymphopenia reflects immune suppression and neurohormonal activation, and is associated with increased susceptibility to infection and heart failure decompensation [27]. Therefore, a low CALLY index represents a composite state of “high inflammation, low nutrition, and impaired immunity,” which may accelerate myocardial remodeling and multiorgan dysfunction, ultimately increasing the risk of MACE. The present findings should be interpreted from the perspective of systemic risk rather than congestion severity alone. Although HFpEF is closely related to haemodynamic congestion and diastolic dysfunction, the CALLY index does not directly measure filling pressure or volume overload. Instead, it integrates inflammatory burden, nutritional reserve, and immune status, thereby capturing a broader systemic vulnerability phenotype associated with adverse outcomes. Therefore, a low CALLY index may identify HFpEF patients with greater systemic inflammation, malnutrition, immune dysregulation, and frailty, which may contribute to poor prognosis independently of, or in addition to, haemodynamic congestion. Accordingly, the CALLY index should not be interpreted as a disease-specific marker of HFpEF inflammatory biology. Importantly, these findings should be interpreted in the context of HFpEF phenotypic heterogeneity and potential diagnostic overclassification. HFpEF is not a single disease entity, and in hospitalized patients with dyspnoea, multimorbidity, modest natriuretic peptide elevation, or extracardiac conditions, clinical diagnosis may be challenging. Because invasive haemodynamic assessment, exercise testing, and objective congestion quantification were not systematically available, our results should be considered applicable to a clinically diagnosed HFpEF cohort rather than to a rigorously phenotyped or haemodynamically confirmed HFpEF population [28]. Emerging evidence also suggests that, after exclusion of major mimicking conditions, HFpEF may often manifest predominantly as exercise intolerance rather than overt congestion at rest [29]. From this perspective, the prognostic value of the CALLY index may reflect systemic vulnerability and impaired physiological reserve, rather than congestion severity alone. Therefore, the CALLY index may help identify clinically vulnerable HFpEF patients, but it should not replace comprehensive phenotypic, haemodynamic, or exercise-based assessment. Rather, it may capture a broader systemic vulnerability phenotype characterized by inflammation, nutritional impairment, immune dysregulation, frailty, and multimorbidity. This interpretation is particularly relevant because CRP may be influenced by respiratory or other extracardiac inflammatory conditions, which are common in patients hospitalized with dyspnoea and may also contribute to modest elevations in NT-proBNP.

From a clinical perspective, effective risk stratification tools for HFpEF remain limited, and traditional models primarily focus on structural and functional parameters without adequately capturing systemic inflammation and nutritional status. The CALLY index, derived from routine blood tests, is simple, cost-effective, and readily available, making it an attractive tool for clinical application. Our findings suggest that the CALLY index may serve as a practical adjunct for identifying high-risk HFpEF patients. For patients with low CALLY index values, closer clinical surveillance and comprehensive assessment of comorbidities, nutritional status, and inflammatory conditions may be warranted. In addition, the CALLY index may have potential as a dynamic biomarker for monitoring treatment response and disease progression.

### Limitations

Several limitations should be acknowledged. First, this was a single-center retrospective study, which may be subject to selection and information bias, and the findings should be considered hypothesis-generating. In particular, atrial fibrillation status was not systematically available in this retrospective cohort and could not be reliably adjusted for. The observational design precludes mechanistic inference. Therefore, a low CALLY index should be interpreted as a marker of clinical vulnerability rather than evidence of a specific inflammatory HFpEF phenotype. In addition, the lack of an external validation cohort limits the generalizability of our findings. Because this was a single-center retrospective study, the predictive performance and optimal cut-off value of the CALLY index may be population-specific. Future multicenter prospective studies with independent validation cohorts are needed to confirm its prognostic value in HFpEF. Second, only baseline CALLY index was evaluated, and its dynamic changes over time were not assessed; longitudinal monitoring may provide additional prognostic information. Third, the sample size and number of events were relatively limited, and residual confounding cannot be entirely excluded despite multivariable adjustment. Fourth, although HFpEF was diagnosed according to ESC guideline criteria, H_2_FPEF or HFA-PEFF scores, invasive haemodynamics, cardiopulmonary exercise testing, exercise stress echocardiography, and objective congestion assessment were not systematically available. Therefore, diagnostic heterogeneity, phenotypic overlap, and potential HFpEF overclassification cannot be excluded. Our findings should thus be interpreted as applicable to a clinically diagnosed hospitalized HFpEF cohort rather than to a haemodynamically confirmed HFpEF population. Fifth, the CALLY index does not directly assess filling pressure, volume overload, or exercise-induced haemodynamic abnormalities. Its prognostic value should therefore not be interpreted as reflecting congestion severity alone, but rather as indicating a broader systemic vulnerability phenotype involving inflammation, malnutrition, immune dysregulation, frailty, and multimorbidity. Future studies with detailed phenotyping and haemodynamic or exercise-based assessment are warranted. Sixth, although clinically evident infection, autoimmune disease, malignancy, and severe hepatic or renal dysfunction were excluded, COPD exacerbation, or other non-cardiac inflammatory conditions could not be fully ruled out. As CRP is a major component of the CALLY index, its prognostic value may reflect systemic vulnerability, frailty, and multimorbidity rather than HFpEF-specific inflammatory activation alone. Finally, the ROC performance may have been overestimated because derivation and testing were performed in the same single-center retrospective cohort without external validation. Moreover, because the CALLY index is derived from CRP, albumin, and lymphocyte count, potential collinearity and redundancy with its individual components should be considered.

## Conclusion

In conclusion, the CALLY index is an independent predictor of long-term MACE in patients with HFpEF and demonstrates a significant nonlinear association with adverse outcomes. As a composite biomarker integrating inflammation, nutritional status, and immune function, the CALLY index provides a simple and potentially valuable tool for risk stratification in HFpEF. Future multicenter prospective studies are warranted to validate its predictive performance and to explore whether CALLY-guided individualized interventions can improve clinical outcomes. 

## Data Availability

The datasets used and/or analyzed during the present study are available from the corresponding author on reasonable request.
